# Contraceptive use and sexual quality of life of patients with thalassemia in Northern Cyprus: a descriptive cross-sectional study

**DOI:** 10.4314/ahs.v23i3.10

**Published:** 2023-09

**Authors:** Sevinc Tastan, Hafize Dogan Ciftci

**Affiliations:** 1 Eastern Mediterranean University, Health Sciences Faculty, Nursing Department, Via Mersin 10, Famagusta, North Cyprus, Turkey; 2 Dr. Burhan Nalbantoglu State Hospital, Nicosia, North Cyprus, Via Mersin 10-Turkey

**Keywords:** Thalassemia, contraceptive, sexual quality of life

## Abstract

**Objective:**

Although contraception methods are an important factor affecting sexual life, no literature has been recorded about the contraceptive methods used and the sexual life of thalassemia patients. The aim of this study is to document the effects of and preferences of contraception methods used in the sexual lives of patients with thalassemia.

**Methods:**

The descriptive and cross-sectional study took place in Northern Cyprus at a Thalassemia Center in a State Hospital. The study sample consisted of 100 thalassemia major or intermedia patients. The data includes descriptive characteristics, the preference of contraception methods used by men and women and Sexual Quality of Life Questionnaires.

**Results:**

Participants learned about contraception methods mostly from social media/internet, and 58.3% of the women and 46.2% of the men did not use any contraception method within the last year. Women's sexual quality of life score was 70.3±19.9 and men's Sexual Quality of Life score was 78.9±20.6. Women having knowledge of contraception methods had higher scores than man (p<0.05). Male patients not having physical exercise had sexual quality of life scores significantly lower than those who followed exercise programs (p<0.05).

**Conclusions:**

Results indicate a significant need to include family planning and sexual health subjects that specifically address thalassemia patients.

## Introduction

Thalassemia, also known as the Mediterranean Anemia, is a major health problem in Cyprus and extends from the Mediterranean to parts of Africa, the Middle East, the Indian subcontinent, Southeast Asia, Melanesia and into the Pacific Islands^1^. For centuries, Thalassemia has been a common disease; when malaria was an endemic disease in the island^2^. In the 1970s, research shows that 15% of the Turkish Cypriots and Greek Cypriots were carriers of beta thalassemia and 10% of the population were alpha thalassemia gene carriers^3^. During this period in Cyprus, Thalassemia Control Programs were beginning to be established. Specifically in Northern Cyprus, thalassemia prevention studies began in the 1980s. Over the past two decades, the birth of babies with thalassemia has been brought under control^2^. Nevertheless, the rate of thalassemia carriers is on an average of 14% in Northern Cyprus, and thalassemia still continues to be an important public health problem^2^.

Hemoglobinopathy prevention programs and thalassemia screening tests are effective in controlling thalassemia baby births, but annually and globally there are still between 300,000 and 400,000 baby births with a serious hemoglobin disorder (23,000 babies with β-thalassemia major)^1^. Prevention programs are accepted as the most successful method to control genetic diseases such as thalassemia by informing society, determining carriers through health screenings, providing genetic counselling, and by using prenatal diagnostic methods to prevent birth defects^4^-^7^. For example, thalassemia can be less traumatic for men and women if they have counselling on contraceptive methods to avoid unwanted pregnancies in carriers and in individuals of high-risk groups. Early detection of a thalassemia during pregnancy may result in the termination of a pregnancy and thereby reduce the physical and emotional trauma experienced by men and women^8^.

The use of effective contraception methods promoted by prevention programs is an indisputable fact in the research. The use of contraception methods is a determining factor that affects men's and women's sexuality and sexual health. Thanks to the use of contraception methods, spouses may become more active in their sexual lives and couples may prevent unwanted pregnancies^9^. Research emphasizes that chronic illnesses and unhealthy lifestyles such as over eating, smoking and drinking alcohol are associated with sexual problems^10^-^12^. Other studies have indicated that there is a negative relationship between chronic illnesses and the sexual quality of life^13^,^14^. Research supports that low self-esteem, unwanted changes in one's physical appearance, and the self-care ability of individuals with chronic illness negatively affects the quality of their sexual lives^15^.

One claim purports a relationship between the use of effective contraception methods and individuals' sexual quality of life. Sexuality issues are of little interest for healthcare professionals so patients have limited access to professional support on the subject. Professional support is often neglected for sexuality issues among people that have chronic diseases. Patients with SQL are also neglected due to the complexity of the types of physical illnesses and treatments available. Moreover, patients tend to hide their problems unless health workers open the subject.

Chronic diseases such as thalassemia and the symptoms of fatigue, excessive stress and anxiety play an important role in sexual dysfunction. In patients with chronic pain and psychological problems, there is a significant reluctance to participate in sexual activity^16^. In the literature, studies with thalassemia patients are usually on their quality of life^17^, ^18^. However, there is no study in the present research about the effects of thalassemia individuals' using contraception methods in their sexual lives. The purpose of this study is to examine the effects of thalassemia patients' contraception method preferences in their sexual lives. It is thought that results of this study will lead to improved family planning consultancy services for thalassemia patients.

## Methods

### Study design

This study was designed as a descriptive and cross-sectional study.

### Participants and Setting

The population of the study began with 180 thalassemia patients who were older than 18 years in age, had been diagnosed with thalassemia and had been provided treatment and follow-ups at the Burhan Nalbantoglu State Hospital's Thalassemia Center in Nicosia, Northern Cyprus. The study inclusion criteria are patients diagnosed with Beta Thalassemia Major and Beta Thalassemia Intermedia; they have active sexual lives, are over 18 years old, are Turkish literate, living in Northern Cyprus and their treatment and follow-ups were conducted at the Centre. Participants who did not accept to participate in the study and were thereby not included (beta-thalassemia minor) did not have an active sexual life, were aged 18 years or younger, not living in Northern Cyprus and they did not have follow-ups but were treated at the Centre. As a result, the sample consists of 109 people that met the inclusion criteria but 9 of the candidates did not agree to participate, leaving 100 participants. The sample represents 91% of the population of 100.

### Instruments

The data was collected using a data collection form and through face-to-face interviews. The data collection form composed of two parts. In the first part, there were demographic questions and questions about contraception methods used. The second part included a Sexual Life Quality Scale for females and a Sexual Life Quality Scale for males.

### The Sexual Quality of Life-Female (SQOL-F)

The SQOL-F was developed by Symonds and colleagues (2005), and the validity and reliability study for the SQOL-F for Turkish women were tested by Tugut and Golbasi (2010)^19^, ^20^. The scale was a 6-point Likert scale and consisted of 18 items. The items total score and reliability coefficient varied between 0.32 and 0.67, and the Cronbach alpha coefficient for internal consistency was 0.8320. In this study, it was determined that the Cronbach alpha reliability coefficient was 0.91.

### Sexual Quality of Life Questionnaire-Male (SQoL-M)

The scale was developed for men by Abraham and colleagues (2008) to assess the quality of sexual life and sexual life disorders^21^. It was a 6-point Likert scale and consisted of 11 items. Moreover, a higher score demonstrated a better sexual quality of life. In the validity and reliability study of the scale for Turkish men, the scale was a one-dimensional structure, and the Cronbach alpha reliability coefficient was 0.75^22^.

### Data Collection

The face-to-face interview data was collected from November 1 to December 20, 2017. The sampling was selected from patients who were at the State Hospital's Thalassemia Center in Nicosia, in Northern Cyprus. Each interview period lasted between 20-30 minutes.

### Ethical Considerations

Before the data collection process, ethical approval was received from the state hospital ethic committees (Nu: YTK.1.01. 138/18-73). The participation was voluntary; in addition, written and verbal consent forms were collected from the participants. For participants' confidentiality, there were no direct identifiers such as name, social insurance number, or health number included the data collection form.

### Statistical Analysis

To analyse the data, the Statistical Package for Social Sciences (SPSS) 24.0 (Chicago, IL, USA) was used. Independent sample t test was used for two independent variables, and variance analysis (ANOVA) was used for more than two independent variables. In the results for ANOVA, when a difference between the categories of the independent variable was found, the Tukey test of the post-hoc test was used to determine the difference of category or categories originated from.

## Results

### Socio-demographic characteristics of the participants

In Table 1, the data collected on the participants' characteristics are given. It was determined that 45.8% of the women who participated in the study were between the ages of 36-45 and 52.1% were married. Of the male patients, 51.9% were between the ages of 36-45 and 50.0% were married. It was determined that 66.7% of females and 76.9% of males had thalassemia major, 54.2% of females and 42.3% of males had thalassemia-diagnosed patients in their family.

**Table 1:** Participant's Characteristics (n=100)

	Female (n1=48)		Male (n2=52)	
	n	%	n	%
**Age (years)**				
≤35	17	35.4	13	25.0
36-45	22	45.8	27	51.9
≥46	9	18.8	12	23.1
**Marital status**				
Married	25	52.1	26	50.0
Unmarried	23	47.9	26	50.0
**Having a child**				
No	26	54.2	32	61.5
Yes	22	45.8	20	38.5
**Smoking**				
No	34	70.8	34	70.8
Yes	14	29.2	18	37.5
**Drinking (Alcohol)**				
No	20	41.7	15	31.3
Yes	28	58.3	37	77.1
**Exercise status**				
Never	18	37.5	16	33.3
Occasionally	23	47.9	25	52.1
Regularly	7	14.6	11	22.9
**Diagnosis**				
Thalassemia major	32	66.7	40	76.9
Thalassemia intermedia	16	33.3	12	23.1
**Family history of Thalassemia**				
No	22	45.8	30	57.7
Yes	26	54.2	22	42.3
**The diagnosis of thalassemia of family members** **(n1=26, n2=22)**				
Thalassemia major	14	29.2	13	25.0
Thalassemia intermedia	12	25.0	9	17.3

### Participants' use of contraceptive methods

Table 2 shows the distribution of using contraception methods by the participants in the study. It was found that 87.5% of the female participants had knowledge about contraception methods. It was determined that 58.3% of female patients did not use any contraception methods in the last year. Of male participants, 67.3% had knowledge about contraception methods. Based on the data, 46.2% of the male patients did not use any contraception methods in the last year. Although not given in the table, female participants had 70.3 ± 19.9 out of 100 from the SQOL-F. When the scores for sexual life quality of male participants were examined, men had 78.9 ± 20.6 out of 100 from the SQoL-M.

**Table 2:** Distribution of Using Contraception Methods

	Female (n=48)		Male (n=52		Total (n=100)	
	n		n	%	n	%
**Having Information for family planning and birth control methods**						
No	6	12.5	17	32.7	23	23.0
Yes	42	87.5	35	67.3	77	77.0
**Source of information (n1=42, n2=35)**						
Physician and other health care professionals	8	19.1	5	14.3	13	13.0
Friends	15	35.7	13	37.1	28	28.0
Social Media/ Internet	19	45.2	17	48.6	36	36.0
**Has had used contraception methods in the last one year**						
No	28	58.3	24	46.2	52	52.0
Yes	20	41.7	28	53.9	48	48.0
**Who used the contraception methods (n1=20, n2=28)**						
Himself/herself	10	50.0	20	38.5	30	30.0
Partner	5	25.0	6	11.5	11	11.0
Both	5	25.0	2	3.9	7	7.0
**Contraception methods used by male (n1=10, n2=22)**						
Condoms	5	50.0	11	50.0	16	16.0
Withdrawal	5	50.0	11	50.0	16	16.0
**Contraception methods used by female**						
Pill	5	33.3	5	62.5	10	10.0
Implant	3	20.0	1	12.5	4	4.0
Bilateral Tubal Ligation	4	26.7	0	0.0	4	4.0
Calendar method	3	20.0	2	25.0	5	5.0

### Participants' Sexual Quality of Life Scores

In Table 3, the sexual quality of life scores was compared according to some of the characteristics of the participants. The average score of the SQOL-F who did not know about the methods of contraception was 53.5 ± 17.3 and those with knowledge were 72.2 ± 19.1 (p<0.05). SQOL-F scores of patients who had knowledge about contraception methods were found to be significantly higher than those who do not have knowledge. For the men, the mean score of the SQoL-M was 80.9 ± 12,0 in the age group of 35 years and under; 83.8 ± 19.4 in the age group of 36-45; and 66.2 ± 26.3 in the age group of 45 years and over (p<0.05). In the advanced analysis, the scale scores of male patients in the age group of 45 years and over were found to be significantly lower than male patients of other age groups. In addition, male patients who did not exercise at all had lower sexual quality of life scores than those exercising sometimes and regularly (p<0.05).

**Table 3:** Comparing participants' sexual quality of life scores according to some characteristics (n=48)

		Female Participants						Male Participants		

	n	M	SD	F/t	p	n	M	SD	F/t	p
**Age (years)**										
≤35	18	73.7	21.9	0.64	0.53	13	80.9	12.0	3.32	0.04*
36-45	22	69.8	20.5			27	83.8	19.4		
≥46	8	64.2	12.5			12	66.2	26.3		
**Having a child**										
	26	66.8	20.3	-	0.27	32	79.8	18.9	0.36	0.72
No				1.11						
Yes	22	73.2	19.1			20	77.6	24.1		
**Exercise status**										
Never	18	67.6	20.4	0.39	0.68	18	68.8	21.4	3.59	0.04*
Occasionally	23	69.9	20.0			23	85.8	18.6		
Regularly	7	75.9	18.9			7	78.8	19.9		
**Diagnosis**										
Thalassemia major	31	72.4	19.1	1.27	0.21	32	81.7	21.4	1.71	0.09
Thalassemia intermedia	16	64.7	20.8			16	70.2	16.3		
**Having information for contraception** **methods**										
No										
	6	53.5	17.3	-	0.03*	17	80.0	23.4	0.25	0.80
				2.25						
Yes	42	72.2	19.1			35	78.5	19.6		

f, ANOVA; t; Independent sample t test

## Discussion

### Participants' use of contraceptive methods

In the present study, factors that were related to using contraception methods and the sexual quality of life for individuals with a diagnosis of thalassemia major and thalassemia intermedia were introduced. The study found that men and women with thalassemia were mostly informed about contraception methods from the Internet and social media. Second, both men and women learned about methods more often from their friends. At a lower rate, they received information from family doctors and other health personnel. Cetisli et al. found that women were more likely to benefit from contraceptive methods, and contrary to the present study that the health professionals played an important role in choosing this method 23. In the present study, it was observed that women and men with thalassemia received little counselling from doctors and health personnel unlike the findings in the study by Cetisli et al.

When health care professionals care for thalassemia patients, they should not only focus on the treatment and care for their illness, but they should also evaluate them holistically, from a physical, mental and social aspect. In a holistic context and especially for individuals in the age range for reproduction, thalassemia patients should be counselled on using contraception methods. Moreover, health workers should inform individuals about contraceptive methods and pregnancy preventive methods or refer them to centers where they may seek help. It is thought that if contraception methods are practiced, unwanted pregnancies may be prevented.

It appears in the present study that more than half of the female patients did not use any contraception methods during the past year, and half of the women who used contraception methods used by herself, one quarter of their spouses used contraception methods. It was found that women who used a contraception method preferred from highest to lowest, an oral contraceptive method, intra uterine device (IUD), tube-ligation and the calendar method. According to the Turkey Demographic and Health Research (2018), the rate of using contraception methods in married women was 70%. The women who used modern contraception methods was at 49%, 21% used traditional methods and the most used method was the withdrawal method 24. There are a limited number of studies in the literature about the rates of using contraception methods by individuals with chronic diseases, and there is no study about thalassemia patients. In a study conducted for young women with chronic illnesses, it was found that 51% of the women did not use a contraception method and 89.4% of them did not receive counselling about contraception methods after being diagnosed^15^. Similar to our study, in the literature, the rate of using preventive contraception methods by women is not at a desired level.

In one study, the findings indicate that 38.5% of men who preferred contraception methods used only the self, and in 11.5% of the population, only the partner used a contraception method. In one study, 91% of the men used one of contraception methods but the percentage of men using modern methods was 66.8% 25. In the same study, 40.3% of the participants used condoms (40.3%) and 33.2% used the withdrawal method (33.2%) 25. The other studies, we found that contraception methods were used at a lower rate in practice, although they are now at a high level of knowledge as our study^25^, ^26^. The use of an effective contraception method by thalassemia individuals will not interrupt their sexual lives both themselves and their spouses; in addition, there will be possibility of having children without psychological, biological and physical exhaustion. These results once again reveal the importance of informing women and men with thalassemia about contraception methods and remind health care professionals about their responsibilities for patients.

### Participants' Sexual Quality of Life Scores

According to Maslow's Hierarchy of Needs theory, sexuality is among the physiological needs in terms of priorities^27^. If an inadequacy or problem in the category cannot be solved, the individual cannot perceive the requirements of the higher level. For example, an individual who cannot satisfy his/her sexual needs is not expected to meet his/her higher needs. In the present study, it was determined that the female patients had 70.3 out of 100 from the SQOL-F, and male individuals had 78.9 out of 100. In one study with healthy subjects, participants had higher scores on sexuality quality of life^22^.

On the other hand, research emphasizes that sexual health is impaired in physically or psychologically chronic patients and patients with physical disabilities^28^, ^29^. In one study, the findings suggest that self-image is affected by organ loss and early menopausal experiences after the surgical procedure and the sexual life is also negatively affected 13. In a study by Mollaoglu et al., two out of three women treated at internal medicine clinics had a sexual dysfunction problem. Sexual disorders are very likely to affect the general health of the individual overall, and we found that the presence of a chronic illness in an individual also affects their sexual life negatively. In other research, results of chronic illnesses, changes in body systems, rapid changes and negative psychosocial states of mind affect people's lives negatively so sexual activity is also negatively affected 30. For this reason, sexual health conditions of individuals with chronic illness should be taken into consideration, and both health care workers and legislators should also take important responsibilities the planning and implementation of the services required.

In this study, the findings indicate that women having knowledge about contraception methods are significantly higher compared to man. There was a significant relationship between age group and exercise status of men, and sexual quality of life scores. Male patients who did not exercise at all were found to have lower sexual quality of life scores (p<0.05). In Ozdemir's study, it is stated that use of contraception methods by men affect women's sexual life and the withdrawal method increases the rate of sexual dysfunction 14. In this study, there is a high incidence of influence in sexual dysfunction by the method of contraception which is used with perceived sexual life and related perception (p<0.05) 14. The results of the present study demonstrate that family planning and counselling services for couples would have a positive effect on their sexual quality of life.

One of the strengths of the study is that it may lead new studies and discussions since it was the first based on the literature review. Another strength is the use of validated questionnaires that determined the sexual quality of life of males and females.

The limitation of the research is that the study was conducted at the only center in Northern Cyprus. The findings may not be generalizable to other populations.

## Conclusion

The results of this study show that the usage rates of contraception methods are at an average level. In this context, the results place significant responsibilities on health professionals who care for patients with thalassemia. First, health care providers need to constantly remind patients that thalassemia is a genetic disorder and the use of modern contraception methods is effective in preventing unwanted pregnancies. Thalassemia patients should be directed to relevant centers for counselling on contraception methods. It is suggested that family planning services should be offered to thalassemia patients in the health departments at hospitals. In future studies, it would be useful to conduct randomized controlled trials to evaluate the effectiveness of family planning education and counselling services for patients with thalassemia. Finally, the repetition of this study with a larger patient population would be beneficial in terms of the generalizability of the results.
